# Health gender gap in Uganda: do weather effects and water play a role?

**DOI:** 10.1186/s12939-022-01769-3

**Published:** 2022-12-05

**Authors:** Emily Injete Amondo, Oliver K. Kirui, Alisher Mirzabaev

**Affiliations:** 1 Center for Development Research (ZEF), University of Bonn, Genscherallee 3, D - 53113 Bonn, Germany; 2 International Food Policy Research Institute (IFPRI), 7th Amarat Street, P.O. Box 474 – 11111, Khartoum, Sudan

**Keywords:** Women health, Water scarcity, Extreme weather events, Time poverty, Rain water harvesting

## Abstract

**Background:**

Vulnerabilities of men and women to adverse health effects due to weather variability and climate change are not equal. Uganda was among the countries in the world most affected by extreme weather events during the last decade. However, there is still limited gendered empirical evidence on the links between weather variability and health and the possible pathways through which these health effects occur. Therefore, this study analyses the effect of weather variability on illness, and the extent to which water collection ‘time burden’ mediates the relationship between weather anomalies and illness among men and women of working age in Uganda. The study also quantifies the health inequalities to be eliminated if resources are equalized.

**Methods:**

Socioeconomic, health and time use data were obtained from the World Bank Living Standards Measurement Studies - Integrated Surveys on Agriculture (LSMS –ISA), combined with high resolution remotely-sensed weather data. Two-parts and non-linear decomposition regression analysis were used on the national representative pooled dataset from the four household survey waves collected between 2009 to 2014, comprising a total of 22,469 men and women aged between 15 and 64 years.

**Results:**

Empirical results show that low rainfall below the long-term mean increased the likelihood of illness by at least 8 and 6 percentage points for women and men, respectively. The indirect effect of low rainfall on illness through water access pathway was estimated at 0.16 percentage points in women. Decomposition results reveal that health inequalities among women and men would have been narrowed by 27–61%, if endowments were equalized.

**Conclusions:**

Strategies that promote women empowerment (such as education, labor force participation, access to financial services and clean water), health adaptation and time poverty reduction strategies (such as rain water harvesting and improved access to quality health care) would reduce gender-based health inequalities in Uganda despite changing climatic conditions.

**Supplementary Information:**

The online version contains supplementary material available at 10.1186/s12939-022-01769-3.

## Background

The importance of gender equality on development outcomes cannot be over emphasized. Apart from enhancing productivity, economic growth and development [1, 2], gender equality also matters for health [3]. In fact, the World Health Organization (WHO) recognizes gender equality as a key social determinant for health [3, 4], interlinked with other development outcomes which impact health, such as improved nutrition and food security [5, 6], lowered fertility and reduced child mortality [4].

Despite significant progress in achievement of gender equality in most countries [7], there still exist significant gender inequalities in many of the low and middle income (LMIC) countries. These countries are also more vulnerable to the changing climate. As a result, they experience acute water shortages for both agricultural and domestic use. For instance, an estimated 400 million people in sub-Saharan Africa (SSA) still have limited access to basic drinking water [8]. Furthermore, the proportion of people with water insecurity is projected to increase in the near future due to climate change [9], translating into more health risks. Limited access to sufficient and safe water partly due to inadequate water infrastructure [10] is acknowledged as one of the three most important factors for poor health in LMIC countries [11]. Therefore, investment in water, sanitation and hygiene (WASH) is important for both health and economic benefits. It is estimated that improvements in WASH could prevent about 10% of disease burden globally, improve productive days and school attendance by additional gains of 320 million and 272 million of healthy days per year, respectively, and provide time savings [12].

Vulnerabilities of men and women to the negative effects of weather variability and climate change on health are not equal. Women are disproportionately affected by (climate) shocks [13, 14] and health risks associated with weather variability [15, 16]. Mainly because of limited access to resources and due to differences in risk exposures and sensitivity [17]. Literature documents that extreme weather events are likely to narrow women’s life expectancy, especially among those with low socio-economic status [16, 17].

There exist several direct and indirect pathways through which climate related health risks occur from a gender perspective. Gender roles in particular, contribute to health disparities among men and women [15], and constitute the indirect pathway through which climate affects health. As “*a gender-based health inequality risk-multiplier*” [14–16], weather variability and climate change may increase the burdens associated with livelihood, household and caring activities [16], thus affecting health of men and women in charge of various activities. In SSA, women provide most of agricultural labor than men [18] and are primary participants when it comes to water collection [19], thus suffer more during periods of water scarcity caused by droughts or shifting rainfall patterns [16]. Weather or climate events may increase distance to water collection points or time spent on water collection activities beyond the WHO/United Nations International Children’s Emergency Fund (UNICEF) Joint monitoring program recommendation of 30 minutes for round trip by for basic water access [20]. This may cause reductions in water consumption per capita, and could be associated adverse health effects, especially among women because of their increased water demands [16].

This paper therefore focuses on linkages between climate variability and health outcomes with a gender perspective, using sex disaggregated national representative dataset and complemented by weather data. We make three major contributions. First, we estimate the total and direct effects of weather variability on health of men and women. Second, we quantify the indirect effect of weather events through water collection time pathway. Third, we determine health gender gap and decompose the sources of these differences, including health seeking behaviors.

There is limited empirical evidence on the above aspects in LMIC countries [21], particularly among the working age populations in rural Uganda. A special focus on this age-group is important given the demographic transition currently experienced in the country where approximately 51.5% of its population are in the working age category, with the projections of continued increase of the share of working age adults in the population until 2070 [22]. Gender inequalities are still persistent in the country - Uganda is ranked in position 65 out of 153 countries with a score of 0.717 in terms of gender parity achievement [7]. High gender inequalities are mainly in rural areas given that the social structure is patriarchal [23].

The rest of the paper is organized as follows; the next section (two) presents the conceptual framework and methods are presented in section three. Descriptive statistics and empirical findings are outlined in section four while section five discusses the findings and section six concludes.

## Conceptual framework

**Fig. 1:**
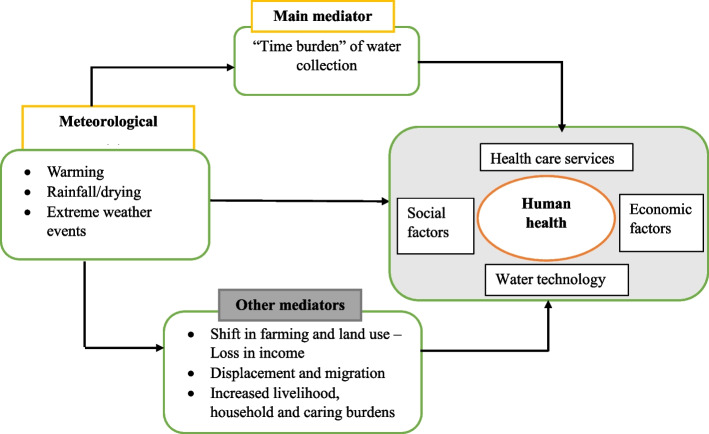
Relationship between climate or weather variability and health, through different pathways

Figure 1 presents the conceptual framework for this study. Our study builds on the framework provided by the World Health Organization [16] on the interactions between climate and health, with a gendered perspective. We highlight both the direct and indirect linkages between exposure to meteorological conditions and health outcomes in Fig. 1. Furthermore, we acknowledge the interactions of different pathways with other non-climatic factors that are major determinants of health. The direct effect of climate on health mainly occur through hazards such as droughts, floods, heatwaves and storms which lead to injuries and mortalities [16, 24, 25]. Additionally, some of infectious diseases outbreaks, resulting from precipitation variations and extremes are classified under the direct pathway [16, 24], even though they occur post-onset. The vulnerability to the abovementioned direct health effects differs by sex and are influenced by social-economic factors.

Indirectly, climate affects health through multiple mechanisms. In general, meteorological changes and extreme weather affect the key determinants of human health which include water, food security, clean air, disease vectors and shelter [14, 16], thus exacerbating already existing diseases. For instance, rising temperatures may increase malaria transmission and pregnancy-induced hypertension [16]. Climate variability may also affect distribution of pathogens responsible for foodborne diseases [26]. Additionally, climate related natural disasters may affect people’s livelihood through loss of assets and income leading to fears, anxiety and other mental health issues [16, 24].

There are different health outcomes associated with lack of access to sufficient and safe drinking water. Water related diseases increase during periods of droughts and floods [27] and water washed diseases resulting from poor hygiene practices are common when there is water scarcity. Taken together, climate events are likely to increase the disease burden attributed water, sanitation and hygiene (WASH) [16, 27]. From a gender lens, it’s important to highlight that women and men have differing needs of water and nutrition in specific periods of their life. Women are at more risk of suffering from health outcomes sensitive to climate such as anemia, malnutrition, micro and macronutrient deficiencies as compared to men [14, 16]. Furthermore, domestic and agricultural tasks performed by women who spent more of their time around house and in the farm, increases their exposure to mosquito breeding sites [14].

In this study, we focus on establishing the indirect effect occurring through the water access pathways. Specifically, time spent on water collection activities which may increase due to weather events with adverse effect on human health [16]. Furthermore, increased water collection time may increase women exposure to contaminated water sources.

Apart from water, sanitation and hygiene related diseases, other negative health impacts resulting from spending more time collecting water and carrying heavy water containers over a long distance from water sources include; increased exposure to heat [14], risk of violence [19, 28], and increased risk of musculo-skeletal illness [16, 19]. Water transport requires physical effort [11, 29], therefore women use a substantial amount of their daily energy intake of about 30% is spent by women fetching water [14, 16], especially during dry seasons, and when water is transported by head loading or hand lifting [29]. Long journeys with heavy pots of water may lead to spinal pain, back pain, head pain and neck pain [11], and potential cumulative damages to muscles [16] and joints, early arthritis and related disabilities due to pressure exerted on the skeletal system [19]. Furthermore, water transport may lead to exhaustion [16], fatigue related injuries [11] and soft tissue damage [19].

Travelling long distances to water source increases the risk of sexual violence that may occur along the way when women travel to fetch water, especially when water collection is carried out in the early mornings or late evenings. Domestic violence may occur at home because of less water collected for household use, and more time spent collecting water compromising other duties [28]. Furthermore, assaults, physical fights and verbal abuse among women over competition for the scarce water resource may occur at water collection points [28], especially during dry seasons. All forms of violence may create anxiety, stress and fear that can consequently lead to mental stress, injuries and sexual health problems [28]. Furthermore, more time spent on water collection activities limits access to health-related inputs such as education and labour income [14, 16], possibility of women neglecting their health care needs [30] and other household activities resulting into more health risks for the entire household [31].

Other mediators such as shift in farming and land use, displacement and migration and other burdens associated with increased livelihood, household and caring activities are presented in the framework because they have adverse effects on health [16], especially in LMIC countries. However, they are out of scope of this study because of data limitations.

## Methods

### Data sources

**Fig. 2:**
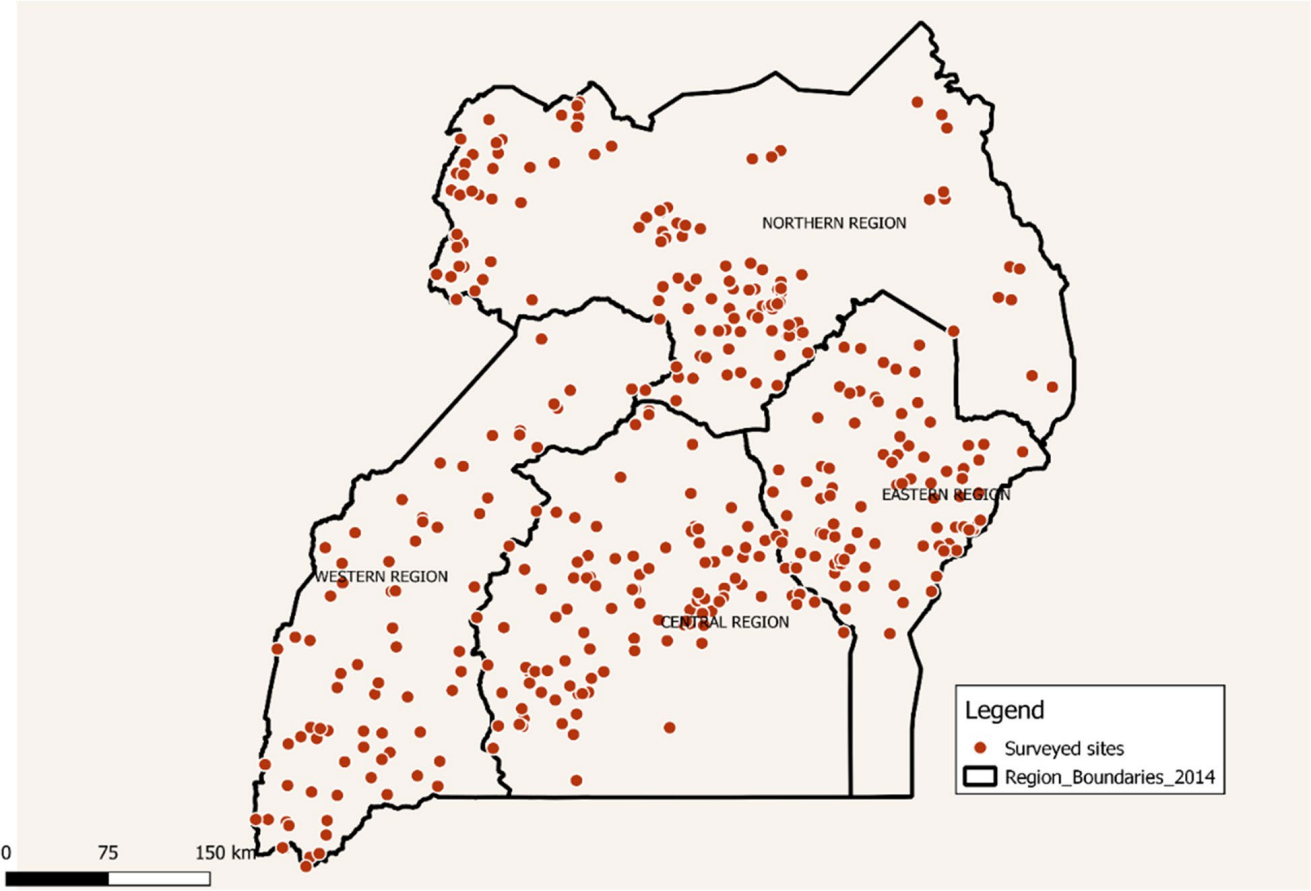
LSMS sampled sites across different regions of Uganda, based on 2009 dataset

The study used three different data sources. Socioeconomic, health and time use data was derived from the LSMS –ISA. Individual and household level data from four waves (2009–2014) were utilized. Specifically, the survey rounds were as follows 2009/2010, 2010/2011, 2011/2012 and 2013/2014. For simplicity we denote the different survey years with the year the survey started 2009, 2010, 2011 and 2013. Sampling was done at household level through two-stage stratified cluster sampling, with samples drawn from all regions and districts of Uganda [32], as shown in Fig. 2. Specific questions on health, education, labor and time use asked for all household members or those above 5 years old. The pooled sample of rural individuals in the working age category (15–64 years) across the four waves was 22,469 men and women.

The georeferenced data in the LSMS enabled us to match each individual in a household with gridded satellite-based temperature and rainfall data within a given enumeration area. Monthly rainfall data was extracted from the Climate Hazards group Infrared Precipitation with Stations (CHIRPS) data version 2, [33] from 1981 to 2014 while the temperature data was from Moderate Resolution Imaging Spectroradiometer-MODIS [34, 35] for the period 2000–2014. These datasets are advantageous because of the high spatial resolution of 0.05^o^ longitude/latitude climate modelling grid [33].

#### Study variables

The main outcome variables are self-reported individual measures of health status, in terms of self-reported morbidity, number of sick days and work day lost due to illnesses or injuries, 30 days prior to the interview. Positive outcome variables are only for the household members whose response was “Yes” to the following question; “*During the past 30 days, did [name] suffer from any illness or injury?*”. We also treated the morbidity dummy variable (Yes/No) as an outcome variable. Even though measures such as functional limitations or clinical measures are preferred as true measures of individual health [36], the survey did not collect this data. However, data on days of work lost due to illness (a proxy of functional limitation and productivity losses) is also included as a measure of health.

The mediator variable is time spent on water collection activities in hours, over the last 7 days prior to the interview. The main explanatory variables are the respective weather variables which comprised of both long-term and short-term variables. Long-term variables include the annual rainfall deviation from the long-term mean (a dummy variable of a dummy variable of (1 = yes) if the annual rainfall deviation from the long-term annual mean (since 1981) was less than 0 mm. For temperature, positive temperature deviation is constructed with (1 = yes) denoting annual average temperature deviation of greater than 0 from the long-term mean temperature. Measures such as negative rainfall deviation or anomalies or shocks for any level of deviation have been used previously by other studies in Uganda context by [37, 38]. Furthermore, following [38] approach, we create additional weather variables for extreme negative rain deviation (a dummy variable of 1 if the negative rainfall deviation values of annual rainfall from the long-term mean annual rainfall fall within the lower range (the 50th percentile and below) while extreme positive temperature deviation is 1 if positive temperature deviation fall within the upper range (50th -100th percentile). Rainfall deviation data is categorized into deciles only for individuals with negative rainfall deviation values while temperature for individuals with positive temperature deviation values. Individuals experiencing extreme negative rainfall had annual rainfall deviation values ranging between -115 mm to – 489 mm with a mean annual deviation of -242 mm while those receiving extreme temperature had annual temperature deviation values of 0.3 °C and above with a mean of 0.76 °C. The distribution of annual and temperature deviation is shown in Fig. S1 in the supplementary materials.

Short term measures weather variables include temperature and rainfall in the month before the interviews. Both variables are continuous and are based on levels rather than deviations. Previous studies on infectious diseases that used such measures in their models include [39, 40].

Other independent variables included in the analysis such as age, marital status, occupation, education, wealth index (using principal component analysis), individual income and health care were guided by literature focusing on the main determinants of health. Given a range of covariates to be included in the models, we tested for multicollinearity using the variance of inflation factor and we report the findings in the results section.

### Empirical strategy

#### Two-part models

Estimation strategies designed to address the problem of limited dependent variables are utilized for this study because the primary outcomes were only reported by a subsample of individuals (approximately a third of the total sample) who were sick.

**Fig. 3:**
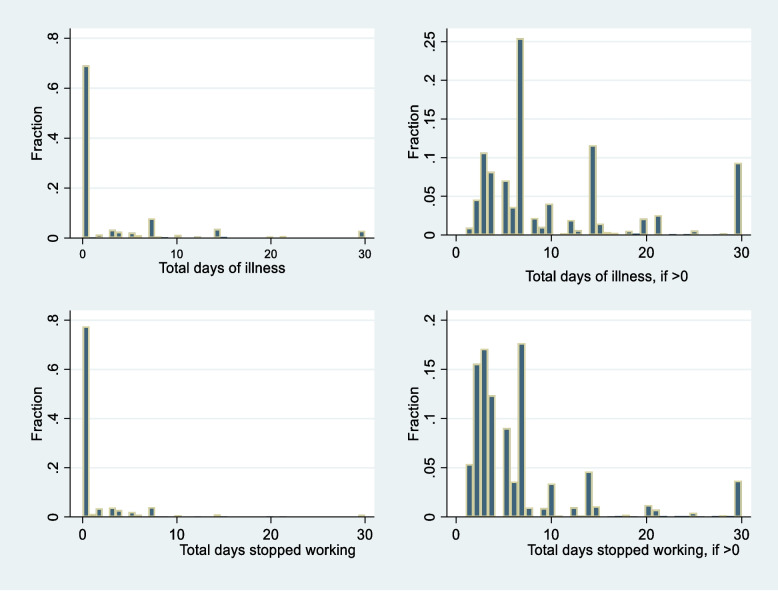
Proportion of individuals with different days of illness and work days lost due to illness

For the rest of the sampled individuals who reported no illness, a positive random variable was not observed. These were substantial number of observations, which were assigned a value of zero, as shown in the left panel of Fig. 3.

Because of the nature and distribution of the outcome variables (large number of zeros, skewness and counts), two-parts model (TPM) commonly used in health economics is adopted. Conceptually, TPM is richer than a one-part model given that it allows decomposition of one random variable into two distinct observed random variables Y > 0 and Y|Y > 0, having different densities [41] thus allowing estimations at both extensive (if any illness or work days lost) and intensive margins (how many days, for those sick). Furthermore, it enables estimation of the overall effect on the whole sample [42, 43], thus making it ideal for use in this study.

In the first part of TPM, the outcome is binary in nature, that is, whether an individual suffered or recorded any day of illness /day of work lost or not. Thus, a logit is used in predicting the probability that an individual has any illness or lost any work day due to illness, and in estimation of the factors that determine the probability of being ill, on the full sample. The general specification predicting the likelihood of a positive outcome in the first part is specified as follows;

1 $$\Pr \left({Y}_{it}>0\right)={\gamma}_0+{\gamma}_1{X}_{1 it}+{\gamma}_2{X}_{2 it}+{\mu}_{it}$$

where *Y*_*it*_ is sick days or days of work lost due to illness for individual *i* in year t, *x*_1*it*_ is a vector of individual or household variables that are determinants of illness such as; age, income, marital status and education. Separate regressions models are estimated for men and women subsamples. *x*_2*it*_ is a vector of variables that represent the different weather variables such as, temperature and rainfall – assumed to be exogeneous and random. Coefficient of interest is *γ*_2_ which measures the total effect of specific weather events on probability of illness. We also control for survey year dummies.

In the second part, a conditional equation is used to model the outcome variable on a subsample of individuals with positive outcomes. The general estimation is specified as;

2 $$E\left({Y}_i|{Y}_i>0\right)={\beta}_0+{\beta}_1{X}_{1 it}+{\beta}_2{X}_{2 it}+{\varepsilon}_1$$

Main coefficient of interest is still (*β*_2_) on the weather variables (*X*_2*i*_) and *ε*_*it*_ is the error term. The generalized linear model (GLM) that can naturally accommodate skewness and is flexible in providing several functional forms and mixed distributions was used [43, 44]. We fit the GLM model using the log link function and gamma as the distribution family, and robust standard errors for statistical corrections. For the overall effect of the two parts model, the expected total days of illness for individual *i* is modelled as individual probability of having days of illness multiplied by the conditional number of days of illness. The general specification that applies also to expected total days of stopped working is specified as follows;

3 $$E\ \left({Y}_{it}\right)=\Pr \left({Y}_{it}>0\right)\ast E\left(\ {Y}_{it}|{Y}_{it}>0\right)=\left({\gamma}_0+{\gamma}_1{X}_{1 it}+{\gamma}_2{X}_{2 it}+{\mu}_i\right)\ast \left(\ {\beta}_0+{\beta}_1{X}_{1 it}+{\beta}_2{X}_{2 it}+{\varepsilon}_1\right)$$

Data analysis was conducted in STATA.

#### Direct, indirect and total effect (mediation) analysis

This study hypothesizes that the total effect of weather events on health operates both directly, and indirectly by influencing the time spent on water collection. Total effect is the sum of both direct and indirect effects and is similar to the effect of a predictor on the outcome when the control or mediator variable is omitted [45] . This effect is derived from the reduced model shown in eq. 1. We use “difference in coefficients methods” to quantify the indirect effect operating through water access pathway. The key variables are weather events with statistically significant coefficients in eq. 1, because they provide evidence for existence of the hypothesized relationship. To measure this mediation, KHB method proposed by Karlson, Holm and Breen that enables cross-model coefficients comparisons of two nested non-linear models, and also enables average partial effects estimations is adopted [46]. This estimation is only conducted at the extensive margin, on the binary response model. The full model after inclusion of the mediator variable (*Z*_*it*_) is estimated as shown below in eq. 5;

4 $$\Pr \left({Y}_{it}>0\right)={\gamma}_0+{\gamma}_1{X}_{1 it}+{\gamma}_2{X}_{2 it}+{\mu}_{it}$$

5 $$\Pr \left({Y}_{it}>0\right)={\alpha}_0+{\alpha}_1{X}_{1i}+{\alpha}_2{X}_{2 it}+{\alpha}_2{Z}_{it}+{\mu}_i$$

Weather variables denoted by *X*_2*it*_ are key variables in both the reduced model (eq. 1 &4) and full model (eq. 5), and the coefficients *γ*_2_ and *α*_2_ represent the total effect and direct effect of specific weather variables respectively. The difference between coefficients of same weather variables (*γ*_2_- *α*_2_) in the two regression equations is a measure of the indirect effect. For interpretation of coefficients on a probability scale, average partial effects are estimated using KHB command with ape option [46]. However, we rely on the logit coefficients for significance test for the effect differences [46].

##### Identification assumptions

The identification conditions for mediation analysis to have a causal interpretation both in observational studies and randomized experiments requires satisfaction of two sequential ignorability assumptions (SIA). These two key assumptions are as follows, 1) The predictor variables (weather variability variables in our case) are conditionally independent of unobserved characteristics, given observed covariates and 2) the mediator variable (water collection time) is also conditionally independent of unobserved characteristics, given other background covariates and predictor variables [45, 47, 48]. However, the second assumption is too strong in many applied settings and not easily testable even in randomized experiments [45, 48] because as much as the treatment variable is randomized, the mediator variable is not, thus creating a self-selection bias.

For the first assumption, even though we do not use experimental data, we argue that this assumption is partly satisfied given that the predictor variables were exogeneous, constructed from remotely sensed data sources as opposed to self-reported measures that are likely to be endogenous. We assume that weather variability is random and uncorrelated with unobserved factors since some of the weather variability measures were deviations from long-term mean. Previous studies that treated weather variability and events as exogeneous variables and potential instruments for causal inference in their studies include [37, 49, 50]. Furthermore, we add as many covariates in our model as possible in order to increase the ignorability of treatment assignment thus strengthening the validity of the outlined identifying assumptions for causal mediation analysis. However, we do not rule out the possibility of existence of unobserved confounders that may affect both the outcome and the mediator variable. Therefore, since we cannot test the second assumption on ignorability of the mediator and do not conduct sensitivity analysis on this non-testable assumption, we do not claim causal effects.

#### Gender gap decomposition analysis on health outcomes

Powers et al. [51] multivariate decomposition method for non-linear models (an extension of Blinder–Oaxaca) is adopted to estimate the gender differences in the health status and explain the source of these differences based on gender-specific factors contributing to observed health inequalities. This method provides estimates for both the overall decomposition and detailed decomposition, thus allowing assessment of each covariate contribution to the different components of the gap [51]. The equations for this decomposition analysis are presented in the Supplementary materials.

## Results

### Descriptive statistics

The socio-economic and weather characteristics of men and women in the working age group are presented in Table S1. On average, women constituted 51% of respondents, and were relatively older (32 years) than men, aged 31 years. Educational attainment levels were significantly lower in women than men, with a difference of approximately 1.5 years. The small gap in education reveal substantial progress made by women in catching up with male education in the recent years. With regards to occupational and income inequalities, a female disadvantage of about 13 percentage points in total for paid work and business activities, and 11 percentage points in income despite a small difference in education attainment provides evidence for gender occupational sorting, partly shaped by the societies [52], especially in LMIC countries.

**Fig. 4:**
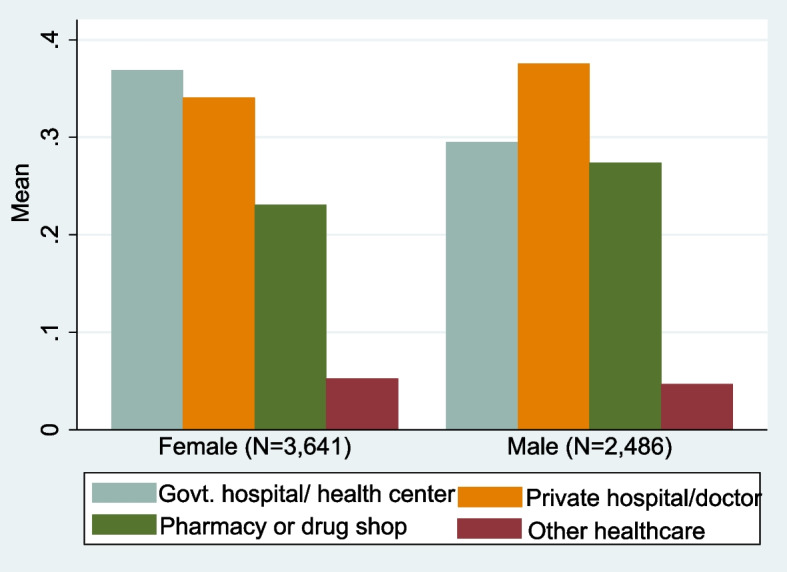
Proportion of men and women consulting different health institutions (only for sick individuals)

More women used treated mosquito nets than men at 42 and 37% respectively with significant differences. Gender differences were also noted on health care seeking behaviors for a sub-sample of individuals who sought consultations. While more women (37%) visited government hospital or health care centers, a significant proportion of men visited private hospitals, private doctors (38%) and pharmacy (27%) than women at 34 and 23% respectively, as shown in Fig. 4. Moreover, health facilities consulted by women were far away (5 km) than those accessed by men (4 km).

**Fig. 5:**
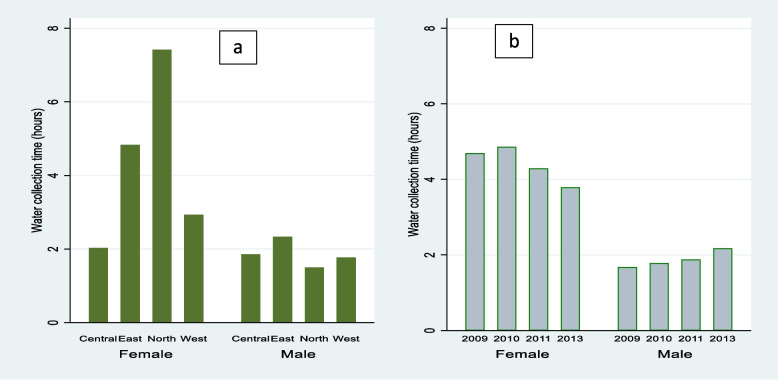
Time spent on water collection, among men and women by region (*a*) and year (*b*)

For intermediate variable, women spent more time collecting water as compared to men, with significant differences. On average, women spent 4.4 hours collecting water while men spent 2 hours as shown in Table S1, with tremendous variations across regions as shown in Fig. 5. Women in the northern region spent more than twice the time spent by women in central and west, despite majority of households (80%) in the north accessing water from improved water sources as shown in Fig. S2 of supplementary materials. Men and women in central spent almost the same number of hours, and overtime there was a slight improvement in men’s time allocation to water activities and reduction in women, which partly signifies progress towards gender equality in division of labour.

On average, 72% of the individuals used improved water sources and 26% used unimproved water sources. Only 4% treated drinking water through both filtering and boiling, 30% practised only boiling, 2% filtered and 61% did nothing in terms of water treatment at home.

#### Health outcome variables

Table 1 reveal women health disadvantage in general. On average, the proportion of women reporting occurrence of illness was significantly higher than men at 36 and 26% respectively. Similarly, approximately a third of the women were unable to continue with their usual activities due to illness. On days of illness and working days lost due to the illness in the 30 days prior to the interview, women reported 3.7 and 1.7 days on average while men reported 2.6 and 1.3 days respectively. The differences in health outcomes between women and men were positive and statistically significant.

**Table 1:** Outcome variable statistics

Variable	Women (*N* = 11,568)		Men N = (10,901)		Difference
	Mean	SD	Mean	SD	
Suffered illness (1 = Yes)	0.357	(0.479)	0.260	(0.439)	0.097***
Days illness (number)	3.734	(6.925)	2.578	(6.008)	1.156***
Stopped working (1 = Yes)	0.262	(0.440)	0.189	(0.391)	0.073***
Days stopped working (number)	1.674	(4.148)	1.283	(4.006)	0.390***

Standard deviations in parentheses *** *p* < 0.01, ** *p* < 0.05, * *p* < 0.1

### Empirical results

#### Effects of weather variables on days of illness (total effect)

The average marginal effects (AME) estimates for multiple weather variables are shown in Table S2.^1^ In general, the logit models indicate that individuals experiencing low rainfall below the long-term mean were more likely to report at least a day of illness as compared to their counterparts,^2^ holding other factors constant. Significant differences were reported in both men and women sub-samples, even though the magnitude was higher in women at −8 percentage points than in men – 6percentage points. Similarly, the coefficients of the overall effect of both parts of the two-parts model were consistently positive and significant, indicating that low rainfall significantly increased days of illness in both men and women, with high effects in women. With regards to extreme negative rain, the magnitude of coefficients at the extensive margin was higher in women at 9 percentage points while the effect in men remain unchanged as shown in columns 2 and 6. However, the overall effect of extreme negative rains in men was more than double the coefficients of negative rain deviation in men. These results imply that extreme weather events have more adverse effects on health, than any level of negative deviation.

Individuals exposed to high annual temperature above the mean reported increased probability of suffering from illnesses. The magnitude of effect was roughly 2 percentage points for both men and women. However, while the overall effect of any level of positive temperature was only significant in men subsample, the overall effect of extreme positive temperature was significant in women sub-sample, with higher coefficient sizes.

Concerning the short-term weather measures, differences in rainfall and temperature experienced in the month before the interview were noted on the coefficient’s signs and significance. For instance, while temperature was positively associated with illness in both men and women, an increase in rainfall was negatively associated with likelihood of illness in men, reducing the probability of illness by 6–7 percentage points. However, the positive and significant coefficients of rainfall quadratic term imply that too much rain maybe detrimental to men health. The overall effect of temperature was significant in both groups, with high magnitude in women.

Other socio-economic determinants of illnesses among men and women include age, education, wealth, use of treated mosquito net, domestic rain water harvesting, water quality, occupation, marital status and income as shown in Table S2.

#### Decomposition of total effect of weather events into direct and indirect effects

Table 2 shows the KHB summary estimates of total, direct and indirect effect of key weather variables (negative rainfall deviation and temperature prior to the interview) through water collection pathway, holding all other covariates constant in Panel A. In general, there were reductions in our key weather coefficients in both men and women subgroups, after introduction of water collection time variable in the full model, providing evidence of the importance of water access pathway in mediating this relationship.

**Table 2:** KHB decomposition results of direct, indirect and total effects of weather variables, through water pathway

	Women				Men			
	Logit		Average partial effects		Logit		Average partial effects	
Variables	Coef.	Std. Err.	Coef.	Std. Err.	Coef.	Std. Err.	Coef.	Std. Err.
	(1)	(2)	(3)	(4)	(5)	(6)	(7)	(8)
**Panel A**								
**Negative rainfall deviation**								
Reduced model (total effect).	0.3890***	0.0665	0.0806***	0.0137	0.3658***	0.0761	0.0648***	0.0134
Full model (direct effect)	0.3814***	0.0665	0.0790***	0.0137	0.3637***	0.0761	0.0644***	0.0134
Difference (Indirect effect)	0.0076**	0.0037	0.0016	.	0.0021	0.0022	0.0004	.
Confounding ratio	1.0198		1.0198		1.006		1.006	
Mediation percentage	1.94		1.94		0.57		0.57	
**Month temperature**								
Reduced model	0.1725***	0.0601	0.0357***	0.0124	0.2478***	0.0718	0.0439***	0.0127
Full model	0.1671***	0.0601	0.0346***	0.0124	0.2462***	0.0718	0.0436***	0.0127
Difference	0.0054	0.0034	0.0011	.	0.0017	0.0021	0.0003	.
Confounding ratio	1.0322		1.0322		1.007		1.007	
Mediation percentage	3.13		3.13		0.67		0.67	
**Panel B**								
**Extreme negative rain**								
Reduced	0.4412***	0.0714	0.0914***	0.0147	0.3632***	0.0776	0.0644***	0.0137
Full	0.4292***	0.0714	0.0889***	0.0147	0.3641***	0.0776	0.0645***	0.0137
Diff	0.0121***	0.0045	0.0025	.	−0.0009	0.0021	−0.0002	.
Confounding ratio	1.0281		1.0281		0.9976		0.9976	
Mediation percentage	2.73		2.73		−0.24		− 0.24	
**Month temperature**								
Reduced	0.1593***	0.0601	0.0330***	0.0124	0.2550***	0.0720	0.0452***	0.0127
Full	0.1541***	0.0602	0.0319***	0.0124	0.2532***	0.0720	0.0449***	0.0127
Diff	0.0052	0.0035	0.0011	.	0.0018	0.0022	0.0003	.
Confounding ratio	1.0337		1.0337		1.0070		1.0070	
Mediation percentage	3.26		3.26		0.70		0.70	
N	11,567		11,567		10,901		10,901	

For women subsample, water collection time mediated the relationship between low rainfall below the long-term mean and probability of illness as well as the relationship between temperature in the month before the interview and likelihood of illness. Exposure to low rainfall increased probability of illness in women by 0.16 percentage points, through the water access pathway. Similarly, an increase in temperature, increased water collection time, which led to a higher likelihood of illness by 0.11 percentage points as shown in columns 1 & 3 of panel A. However, the indirect effect of monthly temperature was insignificant. The KHB ape estimates do not indicate the standard error and significance levels, therefore we rely on KHB logit estimates. The mediation percentages reveal that 2 and 3% of the total effect was due to water time burdens.

Similar results were observed on the extreme negative rain variable where total, direct and indirect effects on health were highly significant in women as shown in Panel B. In fact, the indirect effect of extreme negative rain and the mediation percentage was almost twice the values reported for negative rain deviation.

For men, water collection time mediated the relationship between low rainfall/temperature and probability of illness. These weather variables led to more time allocation on water collection, which increased the likelihood of illness by 0.04 percentage points (indirect effect) as shown in column 5 and 7. However, this mediation effect was insignificant for all of our key weather variables. Only direct and total effects were significant in men.

**Fig. 6:**
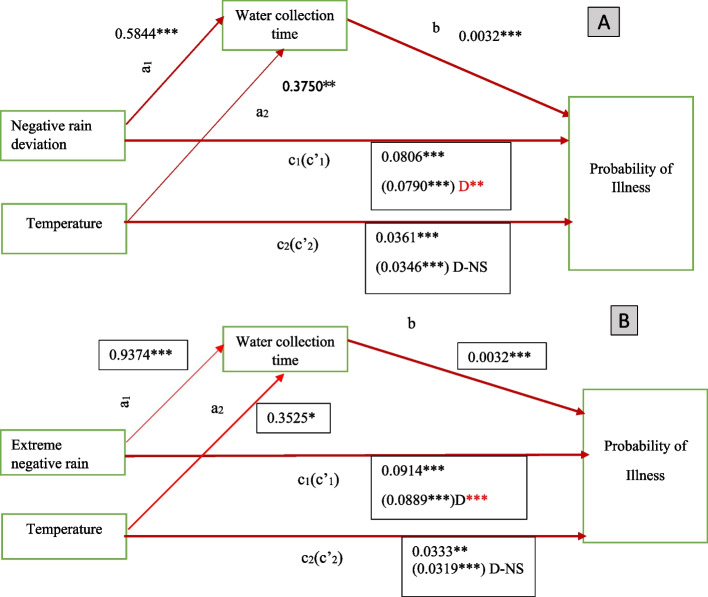
Path diagram with coefficients of total, direct and indirect effects of weather on illness in women (**A**) with negative rain deviation and (**B**) with extreme negative rain. NB: Red lines indicate significant paths while black insignificant. D denotes (indirect effect) *** *p* < 0.01, ** *p* < 0.05, * *p* < 0.1

**Fig. 7:**
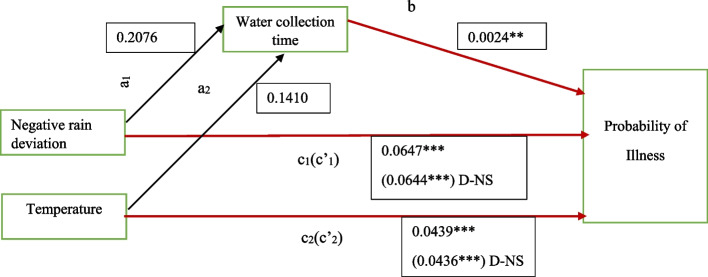
Path diagram showing the total, direct and indirect effects of weather on illness in men

Further results on the coefficient estimates of water collection time on illness in the full model and coefficients of weather variables on water collection time are presented in Figs. 6 and 7,^3^ and Tables S3, S4 and S5 of supplementary materials. The results suggest that key weather events increased water collection time significantly in women by up to 0.9 hours and not in men, while domestic rain water harvesting reduced water collection time in both men and women, with higher magnitudes in women. Similarly, increase in water collection time significantly increased illness in both men and women by 0.32 and 0.24 percentage points respectively. In conclusion, our results support fully the complete mediation process of water collection time in the relationship between negative rain deviation/extreme negative rains and illness in women since all paths (a, b and c) and indirect effect were significant, while mediation process in men was partial given that some of the paths and the difference in coefficients were insignificant. Both the effect of extreme negative rain on water collection time and the indirect effect on illness in women was of higher magnitude as compared to the negative rain deviation as shown in Fig. 6(a) and (b). Improved water source was positively and significantly associated with water collection time in women as shown in Table S4.

#### Summary of mediation analysis

NB: Notes above apply.

#### Effect of weather variables on work days lost due to the illness

Table S6 present two-part estimation results on the gender differentiated effects of weather variables on days an individual stopped working due to the illness. Just like in the case of days of illnesses, holding other factors constant, women and men experiencing low rainfall below the long-term mean were more likely to report illness related work absences by at least 5 percentage points, than those who did not experience low rainfall, as shown in columns 1, 2, 5 and 6. However, the magnitude of effect of long-term low rainfall, especially extreme negative rain was higher in women than in men, with a difference of about 3 percentage points. The overall effects of low rainfall on both parts of the model for the whole sample was positive and significant in both groups, with higher effects in women – 0.5 days of workdays lost. Similarly, too much rainfall in the month before the interview increased the number of work days lost in women by up to 0.6 days.

Exposure to high positive temperature variation increased the probability of work days in both men and women, however, the effects including that of extreme positive temperature effect were insignificant. On the contrary, the effect size of the short-term temperature on likelihood of absence from work was lower and insignificant in women, whereas in men it was positive and significant.

#### Multivariate decomposition results

**Fig. 8:**
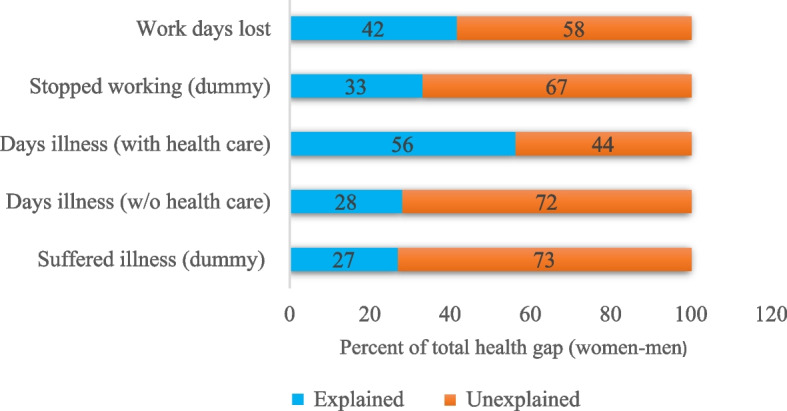
A summary of percentages of explained and unexplained components of the total gender health (without weather extremes)

Table S7 and S8 in the supplementary materials presents results for logistic and negative binomial gender health gap decomposition estimates, for overall and detailed decomposition, without health care variables. The overall decomposition reveals that most of the differences in the health status in terms of prevalence of illness and work days lost between women and men were due to coefficients or differences in effects, thus unexplained. Only 27 and 33% of the gender health gap (suffering illness and stop working) was explained by differences in observed endowments or characteristics between women and men as shown in Fig. 8. Similarly, differences in characteristics accounted for 28 and 42% of the overall women-men health gap in terms of the number of days of illness or workdays lost respectively. Differences in socio-economic variables such as age, education, marital status and temperature contributed to significantly higher proportion of the explained component of health gap. This implies that inequalities in health status between women and men would be eliminated or narrowed if all individuals of both gender groups were of the same age, education and marital status.

**Fig. 9:**
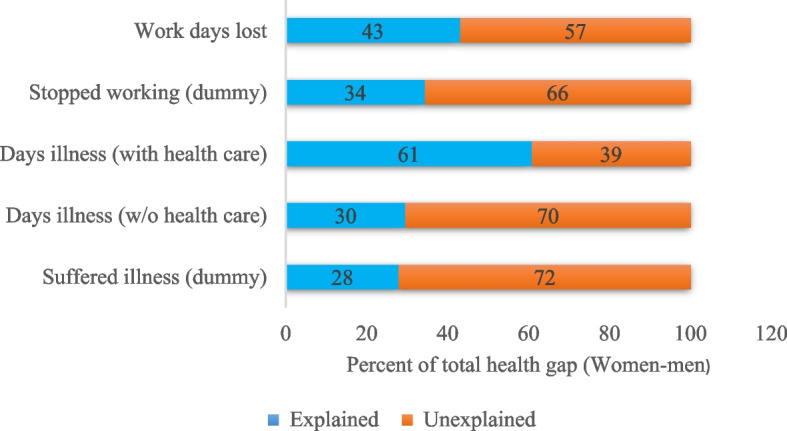
A summary of percentages of explained and unexplained components of the total gender health (*with weather extremes*)

Summary results for decomposition including extreme weather events are shown in Table S8 and Fig. 9. In general, the proportion of explained by differences in observed endowments increased for each health outcome under consideration. For instance, differences in characteristics accounted up to 61% of the total health gap, a difference of about 5% compared to estimates with non-weather extremes.

Controlling for health seeking behaviors in the decomposition analysis, the proportion of gender health inequalities explained by the endowment component increased significantly, as shown in Fig. 8 and Table S9. In particular, the explained health gap in the number of days of illness doubled to 56% as compared to 28% without health care variables. This reveals the importance of health care services, especially distance to the health care centers and places where individuals sought treatment. Contribution of selected individual factors are detailed in Table S9.

## Discussion

This study analyzed the effects of weather anomalies on days of illness and days of work lost due to illness among men and women groups and established the indirect effect through water collection labor burdens. Empirical results showed that low rainfall significantly increased the likelihood of illness of women by 8 percentage points and in men by 6 percentage points. These results are consistent with Lohmann et al. [53] who found out that drought increased the probability of general diseases by about 10 percentage points among individuals. Even though the magnitude of effect in our study was almost similar to Lohmann et al. [53] study, their study did not find any significance differences by gender. Other studies on effects of droughts and mental health found out that prolonged drought was associated with increased likelihood of psychological distress, especially in the rural areas [54], and drought related worries especially among the working individuals [55]. Similarly, droughts were reported to intensify health outcomes among adolescents in rural Ethiopia [56]. On the contrary, Khalili et al. [57] reported decrease in health expenditures for households affected with droughts. Apart from the highlighted health risks, Yusa et al. [58] documented importance of drought on illnesses such as infectious and respiratory diseases, injuries and food/water insecurity related illness. Similarly, a recent study in Uganda reported that low annual rainfall had adverse effect on child health as measured by fever, cough and diarrhea, and fever [59]. Most of the highlighted studies did not establish the effects of drought on different gender categories.

Further results indicate that water collection time fully mediated the relationship between negative rainfall events and illness in women and partially mediated the relationship in men. Full mediation means that occurrence of low rainfall increased water collection time in women, and in turn, both low rainfall and water collection time burden directly affected health outcomes. Low rainfall may result in water scarcity due to decreased water table thus increasing the distance to the nearest water sources as well as increased waiting time at collection points. One study in Uganda, reported that a high proportion of households (85%) in Kasali subcounty spent significantly more time collecting water during drier periods (at least 1 h a day) as compared to wet seasons [60]. More time spent at water source and on hilly roads, especially when carrying a heavy container of water has severe health consequences on women and girls. Men on the other hand are likely to collect water using bicycles and motorcycles which offers them flexibility to fetch water at any time and carrying multiple water containers thus saving time and energy. Furthermore, men are more likely not to queue at the water source, with cases of boys jumping the queue reported by [29].

Other earlier studies in Uganda revealed that women and children suffered more from health complications associated with water collection such as chest pains, fatigue, risk of rape and headaches when conducting water collection activities [29]. Indeed, headache was the second most major symptom reported by approximately 10% of the women in our sample. Other common symptoms such as fever, weakness, coughing, abdominal pain and diarrhea were prevalent in both men and women. Some of the abovementioned symptoms are major signs of acute gastrointestinal illness linked to decreased water availability and quality due to precipitation shortfalls in Uganda [61]. Furthermore, Epstein et al. [59] highlighted water availability as one of the hypothesized mechanisms through which drought or reduced precipitation affects illness in Uganda, especially diarrhea.

Households may also switch the primary water sources between seasons. For instance, almost 20% of households in Uganda switched to a water source with high contamination risk from a low risk water source during dry seasons [62], endangering the health of women who get in close contact with contaminated water sources. Okyere [63] estimates are consistent with our findings where use of improved water sources led to reduction on incidence of illness in males as compared to females. On the contrary, treatment of drinking water through boiling and filtering was associated with lower likelihood of illness in women. These findings imply that different WASH technologies have different health implications in men and women.

Our findings indicate that differences in health seeking behaviors explained a significant proportion of the health gap (over half of the total explained component) at the intensive margin of days of illness. Furthermore, heterogeneity was observed in the places where men and women visited for health care services. Previous studies found that women faced greater barriers in access to healthcare services as compared to men [64].

## Conclusion

Findings in this paper indicate that while both men’s health and women’s health were affected by weather variability, rainfall shortfalls affected health of women more negatively compared to that of men. Moreover, the study provides evidence of the significant role of water access pathway as an important intermediary in the relationship between weather variability and health; especially among women. Weak effects of the water access pathway were reported in men. The differences in access to opportunities and resources, risk exposures and sensitivity between men and women partly explain the differences in overall health and vulnerability to the weather events. Indeed, the study found that up to 60% of health inequalities among women and men would have been narrowed down if endowments were equalized.

Given that women had poorer health than men and were less economically endowed, investment in women’s education and non-farm employment, improving water sanitation and hygiene conditions as well as investment in health adaptation, may aid in reducing the propensity for illness, and the subsequent days of illness or unproductive days and improve the welfare of rural households in Uganda. Low cost water technologies that facilitate household water quantity and safe drinking water, such as domestic rain water harvesting and treatment at either the water source or at home, are recommended.

This study has the following limitations which ought to be considered while interpreting the findings. There was data deficiency on health adaptation measures and on individual level data on other pathways that were of interest to the study. In addition, weather variables were for the enumeration areas as opposed to locations where specific individuals spent time. We do not assess the future gendered health implications under different climate scenarios. Illness measures were captured over a short time period and were based on self-reported symptoms. Data on clinical health measures and inability to do activities of daily living to supplement illness measure were unavailable. Finally, our mediation results are only indicative of the potential effects of weather and mediator variables on health and not causal, therefore they should be interpreted with caution even though they are important.

## Supplementary Information

**Additional file 1.**

## Data Availability

The datasets analyzed in the study are available from the corresponding author on reasonable request.

## Footnotes

1 GLM results on sub-sample of individuals who are sick are available upon request.

2 Probability of reporting at least 1 day of illness and likelihood of illness are used interchangeably throughout the document

3 GLM models are used for Paths A analysis.
