# Supplementary material for: Health gender gap in Uganda: do weather effects and water play a role?
Source: Int J Equity Health. 2022 Dec 5;21:173. doi: 10.1186/s12939-022-01769-3 (PMC9720924; doi:10.1186/s12939-022-01769-3)
Supplement: Supplementary file 1 — Additional file 1. [file 12939_2022_1769_MOESM1_ESM.docx]

**Supplementary materials**

**Health gender gap in Uganda: Do weather effects and water access play a role?**

# **Empirical Strategy**

### **Gender gap decomposition analysis on health outcomes**

Using women subsample as the comparison group and men as the reference category, the overall decomposition of the women-men aggregate gap in health outcome is specified as shown in equation 6 while the final decomposition of the raw aggregate gap expressed as a summation of the weighted total of each factor unique contribution, after weighting is shown in equation 7;

| $\bar{Y}_{w}-\bar{Y}_{m}=\left[ F\left( \bar{X}_{w}\hat{\beta}_{w} \right)-\left( \bar{X}_{m}\hat{\beta}_{w} \right) \right]-\left[ F\left( \bar{X}_{m}\hat{\beta}_{w} \right)-\left( \bar{X}_{m}\hat{\beta}_{m} \right) \right]$ | (6) |
| --- | --- |
| $\bar{Y}_{w}-\bar{Y}_{m}=E+C=\sum_{k=1}^{K} W_{{\Delta X}_{k}}E+\sum_{k=1}^{K} W_{{\Delta X}_{k}}C=\sum_{k=1}^{K} E_{k}+ \sum_{k=1}^{K} C_{k}$ | (7) |

Where $\bar{Y}_{w}-\bar{Y}_{m}$ , is the mean differences in the health outcomes between women and men. The first part $\left[ F\left( \bar{X}_{w}\hat{\beta}_{w} \right)-\left( \bar{X}_{m}\hat{\beta}_{w} \right) \right]$ is due to composition differential between men and women, also known as the explained component given that it is due to the differences in characteristics or endowments (such as age, education, income) between the two groups (Jann, 2008; Powers et al., 2011). The second part $\left[ F\left( \bar{X}_{m}\hat{\beta}_{w} \right)-\left( \bar{X}_{m}\hat{\beta}_{m} \right) \right]$ is known as the coefficient effect or unexplained component which is due to differences in coefficients, behavioral responses or returns. $W_{{\Delta X}_{k}}$ is the decomposition weights while E and C are the explained and unexplained components respectively.

# **Descriptive statistics**


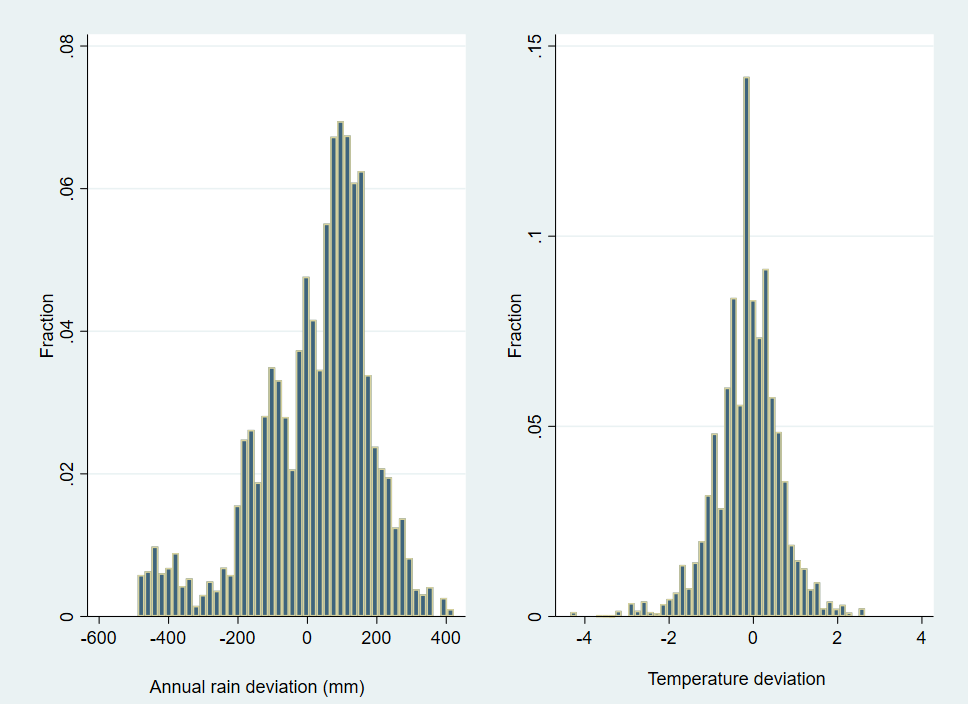


**Fig S1** Distribution of annual rainfall deviation from mean -1981 to survey years (a) and temperature deviations from the mean – 2000 to survey years .

Source: Author elaborations from CHIRPS and MODIS data

**Table S1** Summary statistics of working age individuals, by gender of an individual

| **Category** | **Variable** | **Women**  **(N= 11,568)** | **Men**  **N= (10,901)** | **Difference** |
| --- | --- | --- | --- | --- |
|  |  | **1** | **2** | **3** |
| Socio-economic  Information | Age (years) | 31.971 | 30.757 | 1.214*** |
|  | Education (years) | 5.059 | 6.555 | -1.495*** |
|  | ***Occupation*** |  |  |  |
|  | Salaried /wage (1 = yes) | 0.161 | 0.279 | -0.118*** |
|  | Business (1 = yes) | 0.172 | 0.183 | -0.010** |
|  | Farming (1 = yes) | 0.866 | 0.802 | 0.064*** |
|  | **Income** |  |  |  |
|  | No personal income (1 = yes) | 0.886 | 0.777 | 0.109*** |
|  | Income (1-250000 UGX) | 0.100 | 0.171 | -0.070*** |
|  | Income (250001-750000) | 0.012 | 0.043 | -0.032*** |
|  | Income (>750000) | 0.002 | 0.009 | -0.007*** |
|  | **Marital status** |  |  |  |
|  | Married monogamous (1 = yes) | 0.411 | 0.392 | 0.018*** |
|  | Married polygamous | 0.151 | 0.109 | 0.043*** |
|  | Divorced / Separated | 0.079 | 0.034 | 0.045*** |
|  | Widow/Widower | 0.070 | 0.006 | 0.065*** |
|  | Never married | 0.289 | 0.459 | -0.170*** |
|  | ***Other factors*** |  |  |  |
|  | HH Asset index | -0.483 | -0.460 | -0.024 |
|  | Water harvesting (1=Yes) | 0.014 | 0.014 | 0.000 |
|  | Dependency ratio | 135.2 | 116.3 | 18.93*** |
| Intermediate  variables | Water time (hours) | 4.386 | 1.887 | 2.499*** |
|  | Water quantity (litres) | 63.784 | 64.902 | -1.119 |
| Weather variables | Negative rain deviation (1=yes) | 0.378 | 0.385 | -0.007 |
|  | Extreme negative rain (1=Yes) | 0.191 | 0.191 | 0.000 |
|  | Positive temperature deviation (1=Yes) | 0.411 | 0.414 | -0.003 |
|  | Extreme positive temperature (1=Yes) | 0.247 | 0.247 | 0.000 |
|  | Rainfall (month mm) | 106.9 | 108.3 | -1.309 |
|  | Temperature (month mm) | 29.23 | 29.12 | 0.112** |
| Health care variables | Treated mosquito nets (1=Yes) | 0.423 | 0.367 | 0.056*** |
|  | Distance to health facility^^[[1]](#footnote-1)^^ (Km) | 4.797 | 4.301 | 0.496** |
| Year dummies | Year dummies (2009/10) 1=Yes | 0.251 | 0.252 | -0.002 |
|  | Year dummies (2010/11) 1=Yes | 0.226 | 0.225 | 0.002 |
|  | Year dummies (2011/12) 1=Yes | 0.253 | 0.251 | 0.001 |
|  | Year dummies (2013/14) 1=Yes | 0.270 | 0.272 | -0.002 |

*** p<0.01, ** p<0.05, * p<0.1

**Fig S2** Proportion of individuals collecting water from different water sources

# **Empirical results**

**Table S2** Average marginal effects of logit and two-part models on effect of weather and determinants of illness

|  | Women | | | | Men | | | |
| --- | --- | --- | --- | --- | --- | --- | --- | --- |
|  | Logit | | TPM | | Logit | | TPM | |
|  | (1) | (2) | (3) | (4) | (5) | (6) | (7) | (8) |
| Negative rain deviation | 0.0806*** |  | 0.999*** |  | 0.0647*** |  | 0.313* |  |
|  | (0.0137) |  | (0.205) |  | (0.0134) |  | (0.179) |  |
| Extreme negative rain |  | 0.0914*** |  | 1.177*** |  | 0.0644*** |  | 0.730*** |
|  |  | (0.0147) |  | (0.209) |  | (0.0137) |  | (0.182) |
| Log monthly rain | -0.0212 | -0.0189 | -0.379 | -0.398 | -0.0689** | -0.0619* | -0.566 | -0.560 |
|  | (0.0354) | (0.0354) | (0.502) | (0.500) | (0.0335) | (0.0335) | (0.459) | (0.459) |
| Log rainfall squared | 0.0050 | 0.0048 | 0.071 | 0.074 | 0.0100** | 0.0092** | 0.083 | 0.084 |
|  | (0.0046) | (0.0046) | (0.065) | (0.065) | (0.0043) | (0.0043) | (0.059) | (0.059) |
| Positive temperature | 0.0199** |  | 0.175 |  | 0.0214** |  | 0.244* |  |
|  | (0.0098) |  | (0.142) |  | (0.0093) |  | (0.125) |  |
| Extreme positive temp |  | 0.0282*** |  | 0.376** |  | 0.0091 |  | 0.139 |
|  |  | (0.0110) |  | (0.157) |  | (0.0104) |  | (0.139) |
| Monthly temperature | 0.0361*** | 0.0333** | 0.458** | 0.406** | 0.0439*** | 0.0453*** | 0.330* | 0.333 |
|  | (0.0125) | (0.0125) | (0.185) | (0.184) | (0.0127) | (0.0127) | (0.180) | (0.180) |
| Temperature squared | -0.0005** | -0.0005** | -0.007** | -0.006* | -0.0006*** | -0.0007*** | -0.005 | -0.005 |
|  | (0.0002) | (0.0002) | (0.003) | (0.003) | (0.0002) | (0.0002) | (0.003) | (0.003) |
| Age | 0.004*** | 0.004*** | 0.080*** | 0.079*** | 0.003*** | 0.003*** | 0.054*** | 0.053*** |
|  | (0.000) | (0.000) | (0.006) | (0.006) | (0.000) | (0.000) | (0.006) | (0.006) |
| Education | -0.006*** | -0.006*** | -0.057*** | -0.059*** | -0.006*** | -0.006*** | -0.088*** | -0.089*** |
|  | (0.001) | (0.001) | (0.021) | (0.021) | (0.001) | (0.001) | (0.017) | (0.017) |
| Asset index | -0.008*** | -0.008*** | -0.095*** | -0.090** | -0.008*** | -0.007*** | -0.142*** | -0.139*** |
|  | (0.003) | (0.003) | (0.037) | (0.036) | (0.002) | (0.002) | (0.032) | (0.031) |
| Water harvesting | 0.014 | 0.014 | 0.207 | 0.224 | -0.101** | -0.104** | -1.032* | -1.010* |
|  | (0.037) | (0.037) | (0.536) | (0.537) | (0.042) | (0.042) | (0.568) | (0.566) |
| Improved water source | -0.007 | -0.012 | -0.153 | -0.204 | -0.013 | -0.019** | -0.110 | -0.149 |
|  | (0.010) | (0.010) | (0.141) | (0.141) | (0.009) | (0.009) | (0.126) | (0.125) |
| Other water source | -0.011 | -0.007 | 1.055 | 1.070 | 0.039 | 0.041 | 0.793 | 0.801 |
|  | (0.057) | (0.057) | (0.780) | (0.770) | (0.049) | (0.050) | (0.645) | (0.652) |
| Treated drinking water | -0.054** | -0.056** | -0.490 | -0.497 | -0.007 | -0.007 | 0.127 | 0.106 |
|  | (0.024) | (0.024) | (0.331) | (0.330) | (0.023) | (0.023) | (0.311) | (0.313) |
| Irrigation use | -0.013 | 0.002 | 0.052 | 0.226 | 0.000 | 0.014 | -0.174 | -0.079 |
|  | (0.033) | (0.033) | (0.473) | (0.473) | (0.030) | (0.030) | (0.415) | (0.421) |
| Treated mosquito net | -0.052*** | -0.052*** | -0.664*** | -0.671*** | -0.022 | -0.023 | -0.396** | -0.400** |
|  | (0.015) | (0.015) | (0.213) | (0.213) | (0.015) | (0.015) | (0.195) | (0.195) |
| Salaried /wage | 0.064*** | 0.064*** | 0.656*** | 0.652*** | 0.072*** | 0.074*** | 0.315* | 0.316* |
|  | (0.014) | (0.014) | (0.197) | (0.197) | (0.012) | (0.012) | (0.162) | (0.162) |
| Business | 0.051*** | 0.050*** | 0.246 | 0.230 | 0.047*** | 0.047*** | 0.206 | 0.195 |
|  | (0.011) | (0.011) | (0.156) | (0.156) | (0.011) | (0.011) | (0.146) | (0.145) |
| Farming | -0.029** | -0.028** | -1.084*** | -1.045*** | 0.018 | 0.018 | -0.568*** | -0.582** |
|  | (0.014) | (0.014) | (0.198) | (0.198) | (0.011) | (0.011) | (0.150) | (0.150) |
| Polygamous | 0.002 | 0.000 | 0.089 | 0.081 | -0.024* | -0.024* | -0.152 | -0.159 |
|  | (0.012) | (0.012) | (0.177) | (0.177) | (0.013) | (0.013) | (0.174) | (0.173) |
| Divorced | 0.018 | 0.019 | 0.305 | 0.303 | 0.050** | 0.051** | 0.656** | 0.641** |
|  | (0.016) | (0.016) | (0.227) | (0.227) | (0.021) | (0.021) | (0.263) | (0.262) |
| Separated | 0.040** | 0.041** | 0.613*** | 0.618*** | 0.060 | 0.057 | 0.980 | 0.931 |
|  | (0.017) | (0.017) | (0.233) | (0.233) | (0.048) | (0.048) | (0.601) | (0.595) |
| Never married | -0.088*** | -0.090*** | -0.985*** | -0.997*** | -0.050*** | -0.050*** | -0.502*** | -0.516*** |
|  | (0.014) | (0.014) | (0.204) | (0.204) | (0.014) | (0.014) | (0.188) | (0.188) |
| Income (1- 250000 UGX) | -0.008 | -0.006 | -0.126 | -0.106 | -0.026* | -0.026* | -0.224 | -0.238 |
|  | (0.017) | (0.017) | (0.235) | (0.235) | (0.014) | (0.014) | (0.181) | (0.181) |
| Income (250,001 – 750,000) | -0.090** | -0.091** | -1.423** | -1.476** | -0.073*** | -0.073*** | -0.596* | -0.598* |
|  | (0.042) | (0.042) | (0.648) | (0.646) | (0.023) | (0.023) | (0.316) | (0.315) |
| Income (> 750,000) | 0.023 | 0.022 | 0.463 | 0.487 | -0.020 | -0.023 | -0.523 | -0.548 |
|  | (0.090) | (0.090) | (1.285) | (1.298) | (0.042) | (0.042) | (0.569) | (0.574) |
| Dependency ratio | 0.000 | 0.000 | -0.001 | -0.001 | 0.000 | 0.000 | 0.000 | 0.000 |
|  | (0.000) | (0.000) | (0.001) | (0.001) | (0.000) | (0.000) | (0.001) | (0.001) |
| Year 2010 | 0.005 | -0.001 | 0.162 | 0.184 | -0.003 | -0.022 | -0.389* | -0.221 |
|  | (0.017) | (0.016) | (0.241) | (0.227) | (0.016) | (0.015) | (0.211) | (0.197) |
| Year 2011 | -0.072*** | -0.080*** | -0.782*** | -0.816*** | -0.051*** | -0.070*** | -0.760*** | -0.583*** |
|  | (0.017) | (0.016) | (0.250) | (0.227) | (0.016) | (0.015) | (0.219) | (0.197) |
| Year 2013 | -0.114*** | -0.097*** | -1.386*** | -1.073*** | -0.128*** | -0.126*** | -1.608*** | -1.394*** |
|  | (0.014) | (0.015) | (0.198) | (0.220) | (0.013) | (0.014) | (0.175) | (0.193) |
| 2^nd^ quarter of the year | -0.028** | -0.032 | -0.186 | -0.227 | -0.049** | -0.051*** | -0.475*** | -0.521*** |
|  | (0.014) | (0.014) | (0.205) | (0.205) | (0.014) | (0.014) | (0.184) | (0.184) |
| 3^rd^ quarter of the year | -0.010 | -0.013 | -0.037 | -0.060 | 0.001 | 0.000 | -0.030 | -0.067 |
|  | (0.013) | (0.013) | (0.190) | (0.190) | (0.013) | (0.013) | (0.168) | (0.168) |
| 4^th^ quarter of the year | 0.029** | 0.027 | 0.245 | 0.217 | 0.038*** | 0.037*** | 0.345** | 0.319* |
|  | (0.013) | (0.013) | (0.192) | (0.192) | (0.013) | (0.013) | (0.170) | (0.171) |
| N | 11,567 | 11,567 | 11,567 | 11,567 | 10,901 | 10,901 | 10,901 | 10,901 |

Standard errors in parentheses

*** p<0.01, ** p<0.05, * p<0.1

**Table S3** Average marginal effects results of logit model on the effect of weather and other determinants on probability of illness (Full model), with mediator variable

|  | Women | | |  | Men | | | |
| --- | --- | --- | --- | --- | --- | --- | --- | --- |
| Variables | dy/dx | Std. Err. | dy/dx | Std. Err. | dy/dx | Std. Err. | dy/dx | Std. Err. |
| Negative rain deviation | 0.0790*** | (0.0137) |  |  | 0.0644*** | (0.0134) |  |  |
| Extreme negative rain | - | - | 0.0889*** | (0.0147) | - | - | 0.0645*** | (0.0137) |
| Log monthly rain | -0.0156 | (0.0355) | -0.0134 | (0.0354) | -0.0704** | (0.0335) | -0.0634* | (0.0335) |
| Log rainfall squared | 0.0042 | (0.0046) | 0.0041 | (0.0046) | 0.0102** | (0.0043) | 0.0093** | (0.0043) |
| Positive temperature | 0.0205** | (0.0098) | - | - | 0.0217** | (0.0093) |  |  |
| Extreme positive temp | - | - | 0.0291*** | (0.0110) | - | - | 0.0093 | (0.0104) |
| Monthly temperature | 0.0346*** | (0.0124) | 0.0319*** | (0.0124) | 0.0436*** | (0.0127) | 0.0449*** | (0.0127) |
| Temperature squared | -0.0005** | (0.0002) | -0.0004** | (0.0002) | -0.0006*** | (0.0002) | -0.0006*** | (0.0002) |
| Water collection time | 0.003*** | (0.001) | 0.0028*** | (0.0007) | 0.0023** | (0.0011) | 0.0023** | (0.0011) |
| Age | 0.005*** | (0.000) | 0.005*** | (0.000) | 0.003*** | (0.000) | 0.003*** | (0.000) |
| Education | -0.006*** | (0.001) | -0.006*** | (0.001) | -0.006*** | (0.001) | -0.006*** | (0.001) |
| Asset index | -0.007** | (0.003) | -0.006** | (0.003) | -0.009*** | (0.002) | -0.007*** | (0.002) |
| Water harvesting | 0.018 | (0.037) | 0.017 | (0.037) | -0.099** | (0.042) | -0.101** | (0.042) |
| Improved water source | -0.009 | (0.010) | -0.013 | (0.010) | -0.013 | (0.009) | -0.019** | (0.009) |
| Other water source | -0.007 | (0.057) | -0.003 | (0.057) | 0.039 | (0.050) | 0.041 | (0.050) |
| Treated drinking water | -0.054** | (0.024) | -0.055** | (0.024) | -0.007 | (0.023) | -0.008 | (0.023) |
| Irrigation use | -0.012 | (0.033) | 0.003 | (0.033) | 0.000 | (0.030) | 0.014 | (0.030) |
| Treated mosquito net | -0.053*** | (0.015) | -0.053*** | (0.015) | -0.022 | (0.015) | -0.022 | (0.015) |
| Salaried/Wage | 0.059*** | (0.014) | 0.059*** | (0.014) | 0.073*** | (0.012) | 0.074*** | (0.012) |
| Business | 0.050*** | (0.011) | 0.049*** | (0.011) | 0.047*** | (0.011) | 0.047*** | (0.011) |
| Farming | -0.033** | (0.014) | -0.031** | (0.014) | 0.016 | (0.011) | 0.016 | (0.011) |
| Polygamous | 0.000 | (0.012) | -0.001 | (0.012) | -0.023* | (0.013) | -0.023* | (0.013) |
| Divorced | 0.019 | (0.016) | 0.020 | (0.016) | 0.048** | (0.021) | 0.048** | (0.021) |
| Separated | 0.041** | (0.017) | 0.042** | (0.017) | 0.057 | (0.048) | 0.054 | (0.048) |
| Never married | -0.084*** | (0.014) | -0.086*** | (0.014) | -0.052*** | (0.014) | -0.053*** | (0.014) |
| Income (1- 250000 UGX) | -0.005 | (0.017) | -0.003 | (0.017) | -0.026* | (0.014) | -0.026* | (0.014) |
| Income (250,001 – 750,000) | -0.086** | (0.042) | -0.087 | (0.042) | -0.073*** | (0.023) | -0.073*** | (0.023) |
| Income (> 750,000) | 0.030 | (0.090) | 0.028 | (0.090) | -0.021 | (0.042) | -0.023 | (0.042) |
| Dependency ratio | 0.000 | (0.000) | 0.000 | (0.000) | 0.000 | (0.000) | 0.000 | (0.000) |
| Year 2010 | 0.004 | (0.017) | -0.002 | (0.016) | -0.003 | (0.016) | -0.022 | (0.015) |
| Year 2011 | -0.072*** | (0.017) | -0.080*** | (0.016) | -0.052*** | (0.016) | -0.070*** | (0.015) |
| Year 2013 | -0.111*** | (0.014) | -0.094*** | (0.015) | -0.130*** | (0.013) | -0.127*** | (0.014) |
| 2nd quarter of the year | -0.028* | (0.014) | -0.031** | (0.014) | -0.048*** | (0.014) | -0.050*** | (0.014) |
| 3rd quarter of the year | -0.011 | (0.013) | -0.014 | (0.013) | 0.002 | (0.013) | 0.000 | (0.013) |
| 4th quarter of the year | 0.029** | 0.013 () | 0.027** | (0.013) | 0.038*** | (0.013) | 0.037*** | (0.013) |
| N | 11,567 |  | 11,567 |  | 10,901 |  | 10,901 |  |

**Table S4** Marginal effects results of effect of weather variables on time spent on water collection

|  | Women GLM | | | | Men GLM | | | |
| --- | --- | --- | --- | --- | --- | --- | --- | --- |
|  | Coefficient | Std. Err. | Coefficient | Std. Err. | Coefficient | Std. Err. | Coefficient | Std. Err. |
| Negative rain deviation | 0.5844*** | (0.1755) |  |  | 0.2076 | (0.1344) |  |  |
| Extreme negative rain |  |  | 0.9374*** | (0.2233) |  |  | -0.0330 | (0.1825) |
| Log monthly rain | -1.5584*** | (0.4260) | -1.4972*** | (0.4247) | 1.2852*** | (0.3477) | 1.2785  *** | (0.3476) |
| Log rainfall squared | 0.2267*** | (0.0549) | 0.2221*** | (0.0548) | -0.1601*** | (0.0455) | -0.1597  *** | (0.0456) |
| Positive temperature | -0.4399*** | (0.1354) |  |  | -0.1477 | (0.1031) |  |  |
| Extreme positive temp |  |  | -0.6949*** | (0.1587) |  |  | 0.0804 | (0.1230) |
| Monthly temperature | 0.3750** | (0.1477) | 0.3525** | (0.1496) | 0.1410 | (0.1291) | 0.1364 | (0.1301) |
| Temperature squared | -0.0037 | (0.0024) | -0.0032 | (0.0024) | -0.0022 | (0.0021) | -0.0021 | (0.0021) |
| Age | -0.100*** | (0.006) | -0.101*** | (0.006) | -0.056*** | (0.006) | -0.056*** | (0.006) |
| Education | -0.155*** | (0.021) | -0.155*** | (0.021) | -0.086*** | (0.015) | -0.087*** | (0.016) |
| Asset index | -0.657*** | (0.036) | -0.657*** | (0.036) | 0.006 | (0.027) | 0.002 | (0.027) |
| Water harvesting | -3.211*** | (0.773) | -3.384*** | (0.763) | -2.163*** | (0.561) | -2.175*** | (0.559) |
| Improved water source | 0.483*** | (0.126) | 0.464*** | (0.126) | -0.036 | (0.110) | -0.021 | (0.107) |
| Other water source | -3.493*** | (0.771) | -3.549*** | (0.755) | -0.215 | (0.632) | -0.193 | (0.638) |
| Treated drinking water | -0.789** | (0.348) | -0.781** | (0.354) | 0.154 | (0.253) | 0.116 | (0.252) |
| Irrigation use | -0.641 | (0.413) | -0.489 | (0.427) | 0.156 | (0.394) | 0.175 | (0.388) |
| Treated mosquito net | 0.084 | (0.201) | 0.093 | (0.202) | -0.369** | (0.177) | -0.372** | (0.176) |
| Salaried/Wage | 1.018*** | (0.191) | 0.973*** | (0.193) | 0.063 | (0.165) | 0.063 | (0.162) |
| Business | 0.037 | (0.157) | 0.017 | (0.158) | -0.193 | (0.151) | -0.190 | (0.150) |
| Farming | 2.320*** | (0.215) | 2.376*** | (0.214) | 0.950*** | (0.147) | 0.955*** | (0.146) |
| Polygamous | 0.580*** | (0.177) | 0.597*** | (0.178) | -0.738*** | (0.214) | -0.725*** | (0.215) |
| Divorced | 0.034 | (0.230) | -0.033 | (0.229) | 1.641*** | (0.223) | 1.646*** | (0.219) |
| Separated | -0.428* | (0.249) | -0.443* | (0.247) | 1.949*** | (0.415) | 1.917*** | (0.409) |
| Never married | -1.217*** | (0.164) | -1.253*** | (0.165) | 0.895*** | (0.146) | 0.909*** | (0.146) |
| Income (1- 250000 UGX) | -0.713*** | (0.230) | -0.637*** | (0.234) | -0.043 | (0.184) | -0.036 | (0.180) |
| Income (250,001 – 750,000) | -1.735*** | (0.675) | -1.879*** | (0.644) | -0.294 | (0.327) | -0.254 | (0.336) |
| Income (> 750,000) | -2.890 | (1.811) | -3.036* | (1.818) | -0.246 | (0.509) | -0.250 | (0.509) |
| Dependency ratio | -0.001 | (0.001) | -0.001 | (0.001) | -0.001 | (0.001) | -0.001 | (0.000) |
| Year 2010 | 0.455** | (0.226) | 0.502** | (0.234) | 0.092 | (0.163) | -0.005 | (0.179) |
| Year 2011 | 0.244 | (0.224) | 0.200 | (0.225) | 0.416** | (0.180) | 0.285 | (0.190) |
| Year 2013 | -0.323* | (0.196) | -0.202 | (0.224) | 1.069*** | (0.157) | 1.072*** | (0.185) |
| 2nd quarter of the year | -0.287 | (0.192) | -0.348* | (0.194) | -0.344** | (0.145) | -0.332** | (0.146) |
| 3rd quarter of the year | 0.369** | (0.178) | 0.368** | (0.177) | -0.289** | (0.132) | -0.293** | (0.131) |
| 4th quarter of the year | -0.011 | (0.184) | 0.003 | (0.185) | -0.165 | (0.139) | -0.165 | (0.139) |
| N | 11,567 |  | 11,567 |  | 10,901 |  | 10,901 |  |

**Table S5** Marginal effects results on relationship between time spent on water collection and probability of illness

|  | Women | | Men | |
| --- | --- | --- | --- | --- |
|  | dy/dx | Std. Err. | dy/dx | Std. Err. |
| Water collection time | 0.0032*** | (0.0007) | 0.0024** | (0.0012) |
| Age | 0.005*** | (0.000) | 0.003*** | (0.000) |
| Education | -0.005*** | (0.001) | -0.005*** | (0.001) |
| Asset index | -0.006** | (0.003) | -0.008*** | (0.002) |
| Water harvesting | 0.008 | (0.037) | -0.112*** | (0.042) |
| Improved water source | -0.010 | (0.010) | -0.013 | (0.009) |
| Other water source | 0.000 | (0.057) | 0.045 | (0.050) |
| Treated drinking water | -0.061*** | (0.024) | -0.011 | (0.023) |
| Irrigation use | 0.007 | (0.033) | 0.013 | (0.030) |
| Treated mosquito net | -0.052*** | (0.015) | -0.022 | (0.015) |
| Salaried/Wage | 0.057*** | (0.014) | 0.076*** | (0.012) |
| Business | 0.051*** | (0.011) | 0.049*** | (0.011) |
| Farming | -0.037*** | (0.014) | 0.018 | (0.011) |
| Polygamous | 0.000 | (0.012) | -0.022* | (0.013) |
| Divorced | 0.025 | (0.016) | 0.056*** | (0.021) |
| Separated | 0.045*** | (0.017) | 0.054 | (0.048) |
| Never married | -0.084*** | (0.014) | -0.050*** | (0.014) |
| Income (1- 250000 UGX) | -0.002 | (0.017) | -0.026* | (0.014) |
| Income (250,001 – 750,000) | -0.077* | (0.042) | -0.072*** | (0.023) |
| Income (> 750,000) | 0.033 | (0.090) | -0.018 | (0.042) |
| Dependency ratio | 0.000 | (0.000) | 0.000 | (0.000) |
| Year 2010 | -0.070*** | (0.012) | -0.065*** | (0.011) |
| Year 2011 | -0.152*** | (0.012) | -0.116*** | (0.011) |
| Year 2013 | -0.155*** | (0.012) | -0.163*** | (0.011) |
| 2nd quarter of the year | -0.013 | (0.012) | -0.042*** | (0.012) |
| 3rd quarter of the year | -0.017 | (0.012) | -0.010 | (0.011) |
| 4th quarter of the year | 0.038*** | (0.012) | 0.040*** | (0.011) |
| N | 11,567 |  | 10,901 |  |

**Table S6** Effect of weather variables on days stopped working – AME of the two-part model

|  | Women | | | | Men | | | |
| --- | --- | --- | --- | --- | --- | --- | --- | --- |
|  | Logit | | TPM | | Logit | | TPM | |
|  | (1) | (2) | (3) | (4) | (5) | (6) | (7) | (8) |
| Negative rain deviation | 0.077*** |  | 0.260** |  | 0.052*** |  | 0.202* |  |
|  | (0.013) |  | (0.124) |  | (0.012) |  | (0.113) |  |
| Extreme negative rain |  | 0.089*** |  | 0.541*** |  | 0.059*** |  | 0.432*** |
|  |  | (0.014) |  | (0.124) |  | (0.012) |  | (0.119) |
| Log monthly rain | 0.0328 | 0.0362 | 0.5579* | 0.5383* | -0.0005 | 0.0008 | -0.245 | -0.239 |
|  | (0.034) | (0.034) | (0.304) | (0.302) | (0.032) | (0.032) | (0.300) | (0.300) |
| Log rainfall squared | -0.003 | -0.003 | -0.059 | -0.056 | 0.001 | 0.001 | 0.037 | 0.0370 |
|  | (0.004) | (0.004) | (0.039) | (0.039) | (0.004) | (0.004) | (0.03) | (0.038) |
| Positive temperature | 0.014 |  | 0.087 |  | 0.006 |  | 0.068 |  |
|  | (0.009) |  | (0.085) |  | (0.008) |  | (0.080) |  |
| Extreme positive temp |  | 0.011 |  | 0.113 |  | 0.006 |  | 0.006 |
|  |  | (0.010) |  | (0.094) |  | (0.009) |  | (0.089) |
| Monthly temperature | 0.016 | 0.015 | 0.058 | 0.043 | 0.034*** | 0.034 | 0.230** | 0.233** |
|  | (0.012) | (0.012) | (0.107) | (0.107) | (0.012) | (0.012) | (0.116) | (0.116) |
| Temperature squared | -0.0002 | -0.0002 | -0.0006 | -0.0004 | -0.0005*** | -0.0005*** | -0.0037* | -0.0037** |
|  | (0.0002) | (0.0002) | (0.0017) | (0.0017) | (0.0002) | (0.0002) | (0.0019) | (0.0019) |
| Water harvesting | 0.008 | 0.007 | 0.131 | 0.141 | -0.092** | -0.092** | -0.620 | -0.622 |
|  | (0.034) | (0.034) | (0.298) | (0.297) | (0.040) | (0.040) | (0.414) | (0.409) |
| Improved water source | -0.032*** | -0.037*** | -0.121 | -0.136* | -0.017** | -0.022*** | -0.043 | -0.072 |
|  | (0.009) | (0.009) | (0.082) | (0.082) | (0.008) | (0.008) | (0.079) | (0.079) |
| Other water source | -0.007 | -0.003 | 0.071 | 0.089 | 0.049 | 0.048 | -0.018 | -0.030 |
|  | (0.053) | (0.053) | (0.454) | (0.452) | (0.043) | (0.043) | (0.390) | (0.397) |
| Treated drinking water | -0.044** | -0.045** | -0.091 | -0.105 | 0.002 | 0.002 | 0.280 | 0.277 |
|  | (0.022) | (0.022) | (0.206) | (0.204) | (0.020) | (0.020) | (0.194) | (0.195) |
| Irrigation use | -0.021 | -0.005 | 0.155 | 0.197 | 0.034 | 0.044* | 0.280 | 0.334 |
|  | (0.031) | (0.031) | (0.272) | (0.271) | (0.026) | (0.026) | (0.224) | (0.225) |
| 2^nd^ quarter of the year | -0.024* | -0.028*** | -0.145 | -0.167 | -0.031** | -0.033*** | -0.225* | -0.239** |
|  | (0.013) | (0.014) | (0.122) | (0.122) | (0.013) | (0.013) | (0.118) | (0.118) |
| 3^rd^ quarter of the year | -0.015 | -0.017 | -0.141 | -0.153 | -0.013 | -0.015 | -0.081 | -0.091 |
|  | (0.012) | (0.012) | (0.112) | (0.113) | (0.011) | (0.012) | (0.109) | (0.109) |
| 4^th^ quarter of the year | 0.012 | 0.011 | 0.003 | -0.010 | 0.030*** | 0.028** | 0.104 | 0.093 |
|  | (0.013) | (0.013) | (0.113) | (0.113) | (0.011) | (0.012) | (0.106) | (0.107) |
| Other variables | Yes | Yes | Yes | Yes | Yes | Yes | Yes | Yes |
| Year variables | Yes | Yes | Yes | Yes | Yes | Yes | Yes | Yes |
| Mediator variables | No | No | No | No | No | No | No | No |
| N | 11,567 | 11,567 | 11,567 | 11,567 | 10,901 | 10,901 | 10,901 | 10,901 |

Standard errors in parentheses, *** p<0.01, ** p<0.05, * p<0.1

**Table S7** Multivariate decomposition of women-men gap on days illnesses and days stopped working

| **VARIABLES** | **Logistic** | | | | **Negative binomial** | | | |
| --- | --- | --- | --- | --- | --- | --- | --- | --- |
|  | Suffered illness (dummy) | | Stopped working (dummy) | | Days illness (number) | | Days stopped working (number) | |
|  | Coefficients | Percent | Coefficients | Percent | Coefficients | Percent | Coefficients | Percent |
| **Overall decomposition** | (1) | (2) | (3) | (4) | (5) | (6) | (7) | (8) |
| Characteristics (E) – Explained | 0.0250*** | 26.99 | 0.0229*** | 33.11 | 0.3174*** | 28.07 | 0.1582*** | 41.70 |
|  | (0.0033) |  | (0.0031) |  | (0.0769) |  | (0.0400) |  |
| Coefficients (C) – Unexplained | 0.0675*** | 73.01 | 0.0463*** | 66.89 | 0.8132*** | 71.93 | 0.2211** | 58.30 |
|  | (0.0089) |  | (0.0078) |  | (0.1770) |  | (0.0945) |  |
| Raw difference | 0.0924*** |  | 0.0692*** |  | 1.1305*** |  | 0.3793*** |  |
|  | (0.0084) |  | (0.0073) |  | (0.1710) |  | (0.0917) |  |
| **Detailed decomposition (E)** |  |  |  |  |  |  |  |  |
| Negative rainfall deviation | -0.0006*** | -0.640 | -0.0006*** | -0.800 | -0.0087*** | -0.770 | -0.0030*** | -0.790 |
|  | (0.0001) |  | (0.0001) |  | (0.0022) |  | (0.0010) |  |
| Log month rain | 0.0003 | 0.330 | -0.0005 | -0.670 | 0.0033 | 0.290 | -0.0069 | -1.820 |
|  | (0.0005) |  | (0.0005) |  | (0.0094) |  | (0.0046) |  |
| Log rain squared | -0.0006 | -0.690 | 0.0003 | 0.480 | -0.0076 | -0.670 | 0.0061 | 1.620 |
|  | (0.0006) |  | (0.0005) |  | (0.0108) |  | (0.0052) |  |
| Positive temperature deviation | -0.0001** | -0.070 | -0.0000 | -0.070 | -0.0004 | -0.040 | -0.0001 | -0.030 |
|  | (0.0000) |  | (0.0000) |  | (0.0006) |  | (0.0003) |  |
| Month temperature | 0.0041*** | 4.400 | 0.0018 | 2.560 | 0.0486* | 4.300 | 0.0094 | 2.480 |
|  | (0.0014) |  | (0.0013) |  | (0.0260) |  | (0.0127) |  |
| Temperature squared | -0.0037** | -3.980 | -0.0016 | -2.340 | -0.0452 | -4.000 | -0.0078 | -2.040 |
|  | (0.0015) |  | (0.0014) |  | (0.0278) |  | (0.0137) |  |
| Age | 0.0053*** | 5.770 | 0.0036*** | 5.190 | 0.0891*** | 7.880 | 0.0355*** | 9.360 |
|  | (0.0005) |  | (0.0005) |  | (0.0127) |  | (0.0056) |  |
| Education | 0.0087*** | 9.430 | 0.0084*** | 12.180 | 0.0965** | 8.540 | 0.0443** | 11.680 |
|  | (0.0022) |  | (0.0021) |  | (0.0455) |  | (0.0223) |  |
| Asset index | 0.0002*** | 0.220 | 0.0001* | 0.150 | 0.0021* | 0.180 | 0.0010* | 0.250 |
|  | (0.0001) |  | (0.0001) |  | (0.0011) |  | (0.0006) |  |
| Unimproved water source | -0.0000 | 0.000 | -0.0000 | -0.020 | 0.0004 | 0.040 | 0.0001 | 0.030 |
|  | (0.0000) |  | (0.0000) |  | (0.0005) |  | (0.0003) |  |
| Water harvesting | 0.0000 | 0.010 | 0.0000 | 0.010 | 0.0000 | 0.000 | 0.0001 | 0.020 |
|  | (0.0000) |  | (0.0000) |  | (0.0002) |  | (0.0001) |  |
| Improved water source | -0.0000 | -0.010 | -0.0000 | -0.060 | -0.0006 | -0.050 | -0.0003 | -0.070 |
|  | (0.0000) |  | (0.0000) |  | (0.0006) |  | (0.0003) |  |
| Other water source | 0.0000 | 0.010 | -0.0000 | 0.000 | -0.0003 | -0.020 | 0.0000 | 0.000 |
|  | (0.0000) |  | (0.0000) |  | (0.0004) |  | (0.0002) |  |
| Treated drinking water | -0.0000** | -0.040 | -0.0000* | -0.050 | -0.0005 | -0.040 | -0.0001 | -0.040 |
|  | (0.0000) |  | (0.0000) |  | (0.0003) |  | (0.0001) |  |
| Irrigation use | 0.0000 | 0.010 | 0.0000 | 0.020 | 0.0001 | 0.010 | -0.0000 | -0.010 |
|  | (0.0000) |  | (0.0000) |  | (0.0005) |  | (0.0002) |  |
| Net usage | 0.0045*** | 4.830 | 0.0017* | 2.500 | 0.0495** | 4.380 | 0.0183* | 4.830 |
|  | (0.0010) |  | (0.0009) |  | (0.0195) |  | (0.0094) |  |
| Treated mosquito net | -0.0030*** | -3.210 | -0.0006 | -0.910 | -0.0326* | -2.880 | -0.0145* | -3.820 |
|  | (0.0009) |  | (0.0008) |  | (0.0167) |  | (0.0082) |  |
| Salaried /wage | -0.0076*** | -8.230 | -0.0045*** | -6.560 | -0.0791** | -7.000 | -0.0404*** | -10.660 |
|  | (0.0017) |  | (0.0015) |  | (0.0327) |  | (0.0155) |  |
| Business | -0.0005*** | -0.580 | -0.0001 | -0.210 | -0.0023 | -0.200 | 0.0011 | 0.300 |
|  | (0.0001) |  | (0.0001) |  | (0.0022) |  | (0.0011) |  |
| Farming | -0.0019** | -2.060 | -0.0015* | -2.230 | -0.0572*** | -5.060 | -0.0371*** | -9.780 |
|  | (0.0009) |  | (0.0008) |  | (0.0156) |  | (0.0076) |  |
| Monogamous | -0.0003* | -0.300 | -0.0001 | -0.200 | -0.0037 | -0.320 | -0.0012 | -0.330 |
|  | (0.0001) |  | (0.0001) |  | (0.0031) |  | (0.0015) |  |
| Polygamous | -0.0006 | -0.630 | -0.0004 | -0.570 | -0.0058 | -0.520 | -0.0033 | -0.860 |
|  | (0.0004) |  | (0.0004) |  | (0.0081) |  | (0.0040) |  |
| Divorced | 0.0001 | 0.160 | 0.0001 | 0.090 | 0.0039 | 0.350 | 0.0043 | 1.140 |
|  | (0.0005) |  | (0.0005) |  | (0.0103) |  | (0.0051) |  |
| Separated | 0.0016* | 1.780 | 0.0010 | 1.460 | 0.0161 | 1.420 | 0.0031 | 0.810 |
|  | (0.0008) |  | (0.0007) |  | (0.0172) |  | (0.0082) |  |
| Never married | 0.0151*** | 16.360 | 0.0124*** | 17.950 | 0.2017*** | 17.840 | 0.0985*** | 25.960 |
|  | (0.0022) |  | (0.0021) |  | (0.0407) |  | (0.0208) |  |
| Income (1- 250000 UGX) | 0.0005 | 0.580 | 0.0013 | 1.950 | 0.0112 | 0.990 | 0.0229** | 6.040 |
|  | (0.0012) |  | (0.0011) |  | (0.0229) |  | (0.0115) |  |
| Income (250,001 – 750,000) | 0.0029** | 3.100 | 0.0021* | 3.080 | 0.0418* | 3.700 | 0.0192 | 5.050 |
|  | (0.0013) |  | (0.0012) |  | (0.0240) |  | (0.0121) |  |
| Income (> 750,000) | -0.0002 | -0.170 | 0.0001 | 0.090 | -0.0012 | -0.100 | 0.0057 | 1.500 |
|  | (0.0006) |  | (0.0006) |  | (0.0117) |  | (0.0059) |  |
| Dependency ratio | 0.0001 | 0.070 | -0.0003 | -0.490 | -0.0084 | -0.740 | 0.0011 | 0.300 |
|  | (0.0008) |  | (0.0007) |  | (0.0149) |  | (0.0074) |  |
| Year 2009 | -0.0001*** | -0.080 | -0.0000 | -0.020 | -0.0006* | -0.050 | -0.0002 | -0.060 |
|  | (0.0000) |  | (0.0000) |  | (0.0003) |  | (0.0002) |  |
| Year 2010 | 0.0001*** | 0.110 | 0.0001*** | 0.100 | 0.0015*** | 0.130 | 0.0004** | 0.090 |
|  | (0.0000) |  | (0.0000) |  | (0.0004) |  | (0.0002) |  |
| Year 2011 | -0.0000*** | -0.040 | -0.0000 | -0.010 | -0.0003 | -0.020 | -0.0002* | -0.060 |
|  | (0.0000) |  | (0.0000) |  | (0.0002) |  | (0.0001) |  |
| Year 2013 | 0.0001*** | 0.130 | 0.0001*** | 0.100 | 0.0016*** | 0.140 | 0.0003** | 0.070 |
|  | (0.0000) |  | (0.0000) |  | (0.0003) |  | (0.0001) |  |
| 1^st^ quarter of the year | 0.0000 | 0.010 | 0.0000 | 0.050 | 0.0000 | 0.000 | 0.0003 | 0.080 |
|  | (0.0000) |  | (0.0000) |  | (0.0009) |  | (0.0004) |  |
| 2^nd^ quarter of the year | 0.0003*** | 0.280 | 0.0002** | 0.240 | 0.0029* | 0.260 | 0.0010 | 0.270 |
|  | (0.0001) |  | (0.0001) |  | (0.0016) |  | (0.0008) |  |
| 3^rd^ quarter of the year | 0.0000 | 0.000 | 0.0000 | 0.010 | -0.0000 | 0.000 | 0.0000 | 0.010 |
|  | (0.0000) |  | (0.0000) |  | (0.0001) |  | (0.0000) |  |
| 4^th^ quarter of the year | 0.0002*** | 0.160 | 0.0001*** | 0.130 | 0.0012* | 0.110 | 0.0005 | 0.120 |
|  | (0.0000) |  | (0.0000) |  | (0.0007) |  | (0.0003) |  |
| Observations | 22,469 |  | 22,469 |  | 22,469 |  | 22,469 |  |

**Table S8** Multivariate decomposition of women-men gaps on days illnesses and days stopped working (*with weather extremes*)

| **VARIABLES** | **Logistic** | | | | **Negative binomial** | | | |
| --- | --- | --- | --- | --- | --- | --- | --- | --- |
|  | Suffered illness (dummy) | | Stopped working (dummy) | | Days illness (number) | | Days stopped working (number) | |
|  | Coefficients | Percent | Coefficients | Percent | Coefficients | Percent | Coefficients | Percent |
| **Overall decomposition** | (1) | (2) | (3) | (4) | (5) | (6) | (7) | (8) |
| Characteristics (E) – Explained | 0.0260*** | 28.02 | 0.0239*** | 34.39 | 0.3359*** | 29.62 | 0.1627*** | 43.14 |
|  | (0.0033) |  | (0.0031) |  | (0.0765) |  | (0.0395) |  |
| Coefficients (C) – Unexplained | 0.0667*** | 71.98 | 0.0456*** | 65.61 | 0.7980*** | 70.38 | 0.2145** | 56.86 |
|  | (0.0089) |  | (0.0078) |  | (0.1763) |  | (0.0941) |  |
| Raw difference | 0.0927*** |  | 0.0695*** |  | 1.1339*** |  | 0.3773*** |  |
|  | (0.0084) |  | (0.0073) |  | (0.1711) |  | (0.0916) |  |
| **Detailed decomposition (E)** |  |  |  |  |  |  |  |  |
| Extreme negative rainfall | 0.00004*** | 0.040 | 0.00004*** | 0.060 | 0.0005*** | 0.040 | 0.0002*** | 0.050 |
|  | (0.00000) |  | (0.00001 |  | (0.0001) |  | (0.0001) |  |
| Log month rain | 0.0003 | 0.300 | -0.0005 | -0.740 | 0.0034 | 0.300 | -0.0070 | -1.840 |
|  | (0.0005) |  | (0.0005) |  | (0.0095) |  | (0.0046) |  |
| Log rain squared | -0.0006 | -0.660 | 0.0004 | 0.530 | -0.0078 | -0.690 | 0.0061 | 1.620 |
|  | (0.0006) |  | (0.0005) |  | (0.0109) |  | (0.0053) |  |
| Extreme positive temperature | -0.0000** | -0.020 | -0.0000 | -0.010 | -0.0003** | -0.030 | -0.0001 | -0.020 |
|  | (0.0000) |  | (0.0000) |  | (0.0001) |  | (0.0001) |  |
| Month temperature | 0.0038*** | 4.050 | 0.0016 | 2.290 | 0.0415 | 3.660 | 0.0081 | 2.150 |
|  | (0.0014) |  | (0.0013) |  | (0.0259) |  | (0.0129) |  |
| Temperature squared | -0.0034** | -3.640 | -0.0014 | -2.050 | -0.0381 | -3.360 | -0.0064 | -1.690 |
|  | (0.0015) |  | (0.0014) |  | (0.0278) |  | (0.0139) |  |
| Other variables | Yes |  | Yes |  | Yes |  | Yes |  |
| Mediator variables | No |  | No |  | No |  | No |  |
| Observations | 22,469 |  | 22,469 |  | 22,469 |  | 22,469 |  |

Standard errors in parentheses

*** p<0.01, ** p<0.05, * p<0.1

**Table S9** Multivariate decomposition results for days of illness at the intensive margin (*with and without extremes*).

| VARIABLES | Days illness (number) | | Days illness (number) | |
| --- | --- | --- | --- | --- |
| **Overall decomposition** | Coefficients | Percentage | Coefficients | Percentage |
| Characteristics (E) – Explained | 0.1952*** | 56.22 | 0.2145*** | 60.78 |
|  | (0.0268) |  | (0.0269) |  |
| Coefficients (C) – Unexplained | 0.1520*** | 43.78 | 0.1384** | 39.22 |
|  | (0.0573) |  | (0.0572) |  |
| Raw difference | 0.3472*** |  | 0.3529*** |  |
|  | (0.0594) |  | (0.0594) |  |
| **Detailed decomposition (E)** |  |  |  |  |
|  |  |  |  |  |
| Distance to the health facilities | 0.0492*** | 14.180 | 0.0495*** | 14.020 |
|  | (0.0182) |  | (0.0171) |  |
| Other health care | 0.0055** | 1.580 | 0.0056** | 1.570 |
|  | (0.0027) |  | (0.0026) |  |
| Government hospital/center | 0.0247 | 7.100 | 0.0261* | 7.390 |
|  | (0.0151) |  | (0.0151) |  |
| Private hospital/doctor | -0.0125 | -3.590 | -0.0126 | -3.560 |
|  | (0.0079) |  | (0.0078) |  |
| Pharmacy or drug/local shop | 0.0708*** | 20.400 | 0.0723*** | 20.500 |
|  | (0.0256) |  | (0.0244) |  |
| Weather variables | Yes |  | Yes |  |
| Extreme weather variables | No |  | Yes |  |
| Other variables | Yes |  | Yes |  |
| Year variables | Yes |  | Yes |  |
| Mediator variable | No |  | No |  |
| Observations | 6,971 |  | 6,971 |  |

1. The sample is only for those who incurred some illness and sought consultations [↑](#footnote-ref-1)
